# Development of rice conidiation media for *Ustilaginoidea virens*

**DOI:** 10.1371/journal.pone.0217667

**Published:** 2019-10-24

**Authors:** Yufu Wang, Fei Wang, Songlin Xie, Yi Liu, Jinsong Qu, Junbin Huang, Weixiao Yin, Chaoxi Luo

**Affiliations:** Department of Plant Pathology, College of Plant Science and Technology and the Key Lab of Crop Disease Monitoring and Safety Control in Hubei Province, Huazhong Agricultural University, Wuhan, China; Leibniz-Institut fur Naturstoff-Forschung und Infektionsbiologie eV Hans-Knoll-Institut, GERMANY

## Abstract

Rice false smut, caused by the ascomycete *Ustilaginoidea virens*, is a serious disease of rice worldwide. Conidia are very important infectious propagules of *U*. *virens*, but the ability of pathogenic isolates to produce conidia frequently decreases in culture, which influences pathogenicity testing. Here, we developed tissue media with rice leaves or panicles that stimulate conidiation of *U*. *virens*. Among the tested media, 0.10 g/ml panicle medium was most efficient for conidiation. Whereas, some rice leaf media more effectively increased conidiation than panicle media except 0.10 g/ml panicle medium, and certain non-filtered tissue media were better than their filtered counterparts. Although the conidia induced in rice tissue media were smaller, they were able to germinate on potato sucrose agar medium and infect rice normally. The rice tissue medium is also workable in inducing conidia for conidiation-defective isolates. This method provides a foundation for the production of conidia by *U*. *virens* that will be widely applicable in pathogenicity testing as well as in genetic analyses for false smut resistance in rice cultivars.

## Introduction

Rice false smut (RFS), caused by the ascomycete *Ustilaginoidea virens*, has become one of the most destructive diseases in the majority of rice-growing regions around the world, resulting in severe field losses of rice[1–5] The typical symptom is smut balls informed in rice panicles, the surface of which is covered by an abundance of powdery, dark-green chlamydospores [6–8]. Frequently, sclerotia can be observed on the surface of false smut balls. In addition to yield losses, *U*. *virens* produces ustiloxins that are toxic to human and animals and can inhibit radicle and plumule growth in plant seedlings[9–11].

*U*. *virens* can overwinter with thick-walled chlamydospores and sclerotia, the latter can produce sexual ascospores. Both ascospores and chlamydospores germinate and produce secondary conidia, which are the sources of primary infection in rice [12–14]. Some studies have indicated that *U*. *virens* can infect roots and other rice tissues [15–17]. Nevertheless, numerous inoculation experiments and studies have shown that the majority of *U*. *virens* infections occur at the booting stage [14, 18–20]. Conidia germinate and enter spikelets through the gap between the lemma and palea [21], after that *U*. *virens* hyphae enter and grow intercellularly in the filaments of rice florets during the early stages of infection [18, 19, 22]. Subsequently, the hyphal growth extends to invade the stigmas and styles, enclosing the floral organs [18]. Finally, the hyphal mass extrudes from the lemma and palea, developing into false smut balls.

As one of the sources of primary infection, chlamydospores have been used to carry out artificial inoculation experiments; however, the incidence of infection achieved has been low [23–25]. In separate studies, mixtures of conidia and hyphae were injected into rice panicles at the booting stage, which can improve the incidence of infection significantly [25–27]. Currently, this method is widely used to assess the pathogenicity of *U*. *virens* and the resistance of rice to RFS [28–32]. The production of conidia depends on the time that the isolate has been in culture, temperature, medium and isolates, etc. Up to 10^8^ conidia/ml can be produced after 6 days in potato dextrose broth (PDB) medium and potato sucrose broth (PSB) medium [33]. Previous research has indicated that 2% sucrose-amended PDB medium containing barley seed produces more conidia in a shorter period of time compared with other media [34]. The largest amount of conidia were detected after 6 or 7 days at 26–28°C with shaking at 140 to 210 rpm [35, 36]. In addition, some studies have found that conidia production was related to individual isolate characteristics [37, 38]. We also found that the conidiation capacity varies in different isolates and degrades with increasing numbers of transfers or, for certain isolates, length of time kept in the laboratory.

In this study, we developed tissue media with rice leaves or panicles that could promote the conidiation of *U*. *virens* and was especially useful for stimulating conidiation in isolates with defective conidial production. Although the conidia produced in this medium were small in size, they germinated and infected rice similarly with these produced in normal PSB medium.

## Materials and methods

### *U*. *virens* isolates and rice cultivars

*U*. *virens* strains D32-1, HWD-2 and UV-8 as well as the GFP-labeled strain G2 were used in this study. *U*. *virens* isolates 09-11-1-1 and 09-14-21 were generously provided by Prof. Shiping Wang (Huazhong Agricultural University), both of which showed defective conidiation after being maintained in a laboratory environment for a long period of time (more than 8 years). The Indica rice cultivar Wanxian 98 and the Japonica rice cultivar Huajing 952 were selected for this study.

### Media and culture conditions

At the late booting stage (3–5 days before the heading stage of rice), leaves and panicles of the Indica and Japonica rice cultivars were collected for media preparation. Subsequently, 0.5, 1.5, 3.0 and 5.0 g of leaves or panicles of Indica rice cultivar Wanxian 98 or Japonica rice cultivar Huajing 952 were crushed with a 22, 000 rpm mixer (Midea, model number: MJ-250BP01B) for 1 min in 40 ml distilled water respectively. Then they were used directly (unfiltered) or filtered with three layers of gauze to prepare media with the final concentrations of 0.01, 0.03, 0.06 and 0.10 g/ml respectively, and the media were autoclaved at 121°C for 30 min.

### Conidiation tests in different media

For conidiation tests, the strain G2 was incubated on PSA (potato sucrose agar: 200 ml of juice from 200 g of potato, 20 g of sucrose, and 15 g of agar per liter) medium at 27°C for 10 days, two 5-mm mycelial plugs were taken from the periphery of colony using a cork borer and incubated in 50 ml of PSB (PSA without agar) medium or liquid rice tissue media at 27°C with shaking at 160 rpm. The production of conidia was investigated from 3^rd^ to 7^th^ day using a hemacytometer. The conidiation from the filtered rice tissue media were compared with unfiltered ones at the 7^th^ day post-incubation (DPI). The conidiation in 0.06 g/ml leaf media were compared with 0.06 g/ml panicle media at 7 DPI. The conidiation in 0.06 g/ml of Indica rice leaf (IRL) medium were compared with 0.06 g/ml of Japonica rice leaf (JRL) medium at 7 DPI. Three replicates were observed for each treatment, with three droplets of each suspension examined per replicate. The experiment was conducted twice. The conidiation in different concentrations of rice tissue media and PSB medium were analyzed with SPSS at *P = 0*.*05*.

### Measurement of conidia size and germination and mycelial growth

To determine conidia size, leaves or panicles from Wanxian 98 or Huajing 952 at late booting stage were collected to make 0.06 g/ml rice tissue media and PSB medium was selected as control. Strain G2 was shaken in liquid media for 7 days and filtered through four layers of gauze to collect its conidia. For each treatment, 100 conidia were measured at two perpendicular directions under a microscope. For the germination test, conidia were harvested by centrifugation at 7, 000 rpm and adjusted to a concentration of approximately 1.0×10^5^ conidia/ml. The conidia were spread on the surface of PSA or WA (water agar: 15 g of agar per liter) plates, and the germination rate was investigated at 12, 24, 36 and 48 h respectively. For each treatment, 100 conidia were measured per replicate and three replicates were carried out. Each experiment was conducted twice. For mycelia growth, 5-mm mycelial plugs were removed from the margins of a colony and placed in the center of PSA, WA and 0.06 g/ml rice tissue media solidified with 2% agar plates. The plates were incubated at 27°C in the dark for three weeks, and two perpendicular colony diameters were measured. Five replicates were performed for each treatment, and the experiment was conducted three times.

### Pathogenicity assay

After 7 days of incubation of strain G2 in 0.06 g/ml IRL medium with shaking, conidia were collected and the concentration was adjusted to 1.0×10^6^ conidia/ml for inoculation as following three treatments: (I) the conidia were collected and diluted with water, (II) the conidia were collected and diluted with PSB, (III) the conidia were collected and cultured in 50 ml of PSB medium at 27°C with shaking at 160 rpm for 8–12 h for germination, and then conidia were collected again and diluted with PSB medium. The inoculation was performed as described previously [18]. In brief, approximately 2 ml of each conidia (1.0×10^6^ conidia/ml) suspension was injected into a single rice panicle at the late booting stage. Inoculated plants were kept in a 27°C greenhouse with 90–100% relative humidity (RH) for 7 days. Then, they were placed at 27°C and 80% RH until rice false smut symptoms appeared. The number of false smuts were recorded and analyzed. Twelve rice panicles were inoculated for each treatment, the experiments were conducted three times.

### Investigation of conidia production of conidiation-defective isolates

The conidiation-defective isolates D32-1, HWD-2, UV-8, 09-11-1-1 and 09-14-21 were selected for conidiation tests, two 5-mm mycelial plugs were taken from the periphery of a 10-day-old colony of each isolate using a cork borer, and incubated in 50 ml of PSB medium or rice tissue media with the concentration of 0.06 g/ml IRL medium at 27°C with shaking at 160 rpm. The amounts of conidia were counted 3^rd^ to 7^th^ day using a hemacytometer. Three replicates were examined for each treatment, with three droplets of each suspension per replicate. The experiments were conducted twice.

## Results

### Rice tissue media promoted the conidiation of *U*. *virens*

To develop a medium that promotes *U*. *virens* conidiation, different rice tissue media were produced to investigate whether rice tissue can promote the conidiation of *U*. *virens*. Conidiation of strain G2 was evaluated at 3, 4, 5, 6 and 7 DPI. As shown in Table 1, compared with PSB medium, Indica rice Wanxian98 leaves or panicles media induced more conidia at individual time points. These results indicated that rice tissue could promote conidia production. Among the four concentrations, in general, higher rice tissue concentrations induced more conidia except for the medium with 0.10 g/ml IRL, which showed fewer conidia compared with other concentrations of IRL (Table 1). It is possible that too much fibrous tissue in the 0.10 g/ml IRL medium hindered the smooth shaking that is important for conidia production.

**Table 1:** Conidia production of *U*. *virens* strain G2 in Indica rice Wanxian 98 tissue media.

Medium^y^	Conidiation (×10^6^ conidia/ml)^x^				
	3 DPI	4 DPI	5 DPI	6 DPI	7 DPI
PSB	0.000±0.000b	0.022±0.022b	0.367±0.367c	1.544±0.953c	1.911±0.946c
0.01 g/ml IRL	0.592±0.196a	4.092±0.497a	7.558±0.509b	9.642±0.582b	9.756±0.563b
0.03 g/ml IRL	0.253±0.185ab	4.922±1.829a	24.531±2.655a	32.183±2.657a	34.014±2.754a
0.06 g/ml IRL	0.094±0.039b	1.092±0.505b	11.144±3.621b	32.931±3.847a	38.964±4.741a
0.10 g/ml IRL	0.000±0.000b	0.036±0.015b	0.131±0.048c	0.217±0.078c	0.447±0.125c
PSB	0.000±0.000b	0.022±0.022b	0.367±0.367b	1.544±0.953d	1.911±0.946d
0.01 g/ml IRLF	0.003±0.003b	0.150±0.056a	1.444±0.198b	2.325±0.327d	2.661±0.649d
0.03 g/ml IRLF	0.011±0.006b	0.078±0.041a	2.089±0.675b	11.278±2.128c	16.372±1.416c
0.06 g/ml IRLF	0.056±0.013a	0.428±0.260a	6.681±1.504a	19.350±1.414b	22.914±1.405b
0.10 g/ml IRLF	0.008±0.006b	0.178±0.053a	5.692±1.826a	25.231±3.362a	32.878±2.940a
PSB	0.000±0.000b	0.022±0.022b	0.367±0.367b	1.544±0.953c	1.911±0.946d
0.01 g/ml IRP	1.303±0.462a	2.689±0.682b	3.089±0.520b	3.639±0.541c	3.675±0.633cd
0.03 g/ml IRP	0.283±0.159b	2.336±0.677b	5.667±1.202b	8.389±1.858c	7.939±1.056c
0.06 g/ml IRP	0.581±0.476ab	7.939±2.135a	27.236±3.534a	27.683±2.080b	28.853±1.305b
0.10 g/ml IRP	0.025±0.025b	3.569±1.853b	27.408±4.694a	45.072±5.903a	40.250±2.769a
PSB	0.000±0.000b	0.022±0.022b	0.367±0.367c	1.544±0.953c	1.911±0.946c
0.01 g/ml IRPF	0.058±0.027a	0.869±0.273ab	1.903±0.383bc	1.806±0.404c	1.661±0.397c
0.03 g/ml IRPF	0.044±0.022ab	0.839±0.499ab	3.250±0.462b	4.633±1.171c	4.536±0.620c
0.06 g/ml IRPF	0.044±0.025ab	2.925±1.925a	10.397±0.544a	24.253±1.071a	27.544±1.113a
0.10 g/ml IRPF	0.006±0.006ab	0.328±0.153ab	7.917±1.917a	17.228±2.699b	21.288±2.107b

^x^ Data shown are the means of two independent experiments, reported as the mean±standard error. Values followed by the same letters within the same column for the same media with different concentrations of rice tissue are not significantly different based on one-way ANOVA with LSD tests performed with SPSS at *P = 0*.*05*. Data were logarithm-transformed before analysis. The same PSB culture was used as the control for the different rice tissue media.

DPI: days post incubation.

^y^ PSB: potato sucrose broth; IRL: Indica rice leaf; IRLF: Indica rice leaf filtrate; IRP: Indica rice panicle; IRPF: Indica rice panicle filtrate.

The unfiltered or filtered rice leaves media were also evaluated. The number of conidia from IRL media was compared with that from IRL filtrate (IRLF) media at 7 DPI. Results showed that more conidia were produced in IRL media at concentrations of 0.01 (*P* = 8.873×10^−6^), 0.03 (*P* = 7.383×10^−4^) and 0.06 g/ml (*P* = 1.755×10^−2^). However, at 0.10 g/ml, fewer conidia were produced in the IRL medium compared with the IRLF medium, most likely because too much rice leaf tissue was present (*P* = 1.071×10^−4^). The similar results were also observed at 3, 4, 5 and 6 DPI.

Considering that *U*. *virens* infects the rice panicle, panicle tissues were also selected to prepare media for conidiation testing. The results of Indica rice Wanxian98 panicle (IRP) media were similar to these of the IRL media at 7 DPI, with the exception of the 0.10 g/ml IRP medium that induced more conidia production (*P* = 4.061×10^−4^), likely because there is less fibrous tissue in rice panicles compared to leaves. Four concentrations of IRP and IRP filtrate (IRPF) media were also compared. More conidia were observed in the IRP media, except for 0.06 g/ml media which did not show significant difference between the IRP and IRPF media (*P* = 0.463).

For the different tissues, compared with IRP, the IRL media induced more conidia at 0.01 (*P* = 3.218×10^−5^) and 0.03 g/ml (*P* = 7.818×10^−5^) at 7 DPI, but no significant difference was observed at 0.06 g/ml (*P* = 0.086). However, at 0.10 g/ml, the IRLs media induced fewer conidia compared with the IRP media (*P* = 2.867×10^−5^).

Similar results were observed when tissues of Japonica rice cultivar Huajing 952 were used. Generally, the rice tissue media induced more conidia, except for the 0.01 g/ml Japonica rice leaf filtrate (JRLF) medium. Compared with JRLF media, JRL media induced more conidia at 0.01 (*P* = 4.608×10–4), 0.03 (*P* = 2.464×10^−5^) and 0.06 g/ml (*P* = 6.709×10–7), and no significant difference was observed at 0.10 g/ml (*P* = 0.340) at 7 DPI. JRP media induced more conidia than JRPF media at 0.01 (*P* = 3.244×10–2), 0.03 (*P* = 7.811×10^−4^), 0.06 g/ml (*P* = 1.910×10–2) and 0.10 g/ml (*P* = 1.006×10–4) at 7 DPI. For different tissues, compared with Japonica rice panicle (JRP) medium at 7 DPI, Japonica rice leaf (JRL) medium induced more conidia at 0.01 (*P* = 3.477×10^−2^), 0.03 (*P* = 8.275×10^−5^) and 0.06 g/ml (*P* = 2.459×10^−2^), but 0.10 g/ml JRP medium induced more conidia (*P* = 1.418×10^−5^), likely because there is less fibrous tissue in rice panicles compared to leaves (Table 2).

**Table 2:** Conidia production of *U*. *virens* strain G2 in Japonica rice Huajing 952 tissue media.

Medium^y^	Conidiation (×10^6^ conidia/ml)^x^				
	3 DPI	4 DPI	5 DPI	6 DPI	7 DPI
PSB	0.000±0.000b	0.022±0.022c	0.367±0.367d	1.544±0.953d	1.911±0.946e
0.01 g/ml JRL	0.486±0.128a	3.347±0.359a	5.544±0.196c	6.886±0.580c	7.275±0.774d
0.03 g/ml JRL	0.097±0.032b	2.442±0.578ab	14.847±1.002b	25.942±1.584b	25.252±1.326b
0.06 g/ml JRL	0.103±0.033b	2.236±0.461b	31.794±2.394a	38.722±2.351a	36.789±1.511a
0.10 g/ml JRL	0.053±0.028b	0.467±0.183c	2.128±0.744cd	5.050±1.199cd	12.150±0.898c
PSB	0.000±0.000b	0.022±0.022b	0.367±0.367b	1.544±0.953b	1.911±0.946b
0.01 g/ml JRLF	0.011±0.008b	0.206±0.134b	0.758±0.196b	1.658±0.069b	1.778±0.193b
0.03 g/ml JRLF	0.081±0.023a	2.608±0.690a	4.769±0.663a	5.850±0.562a	7.989±1.739a
0.06 g/ml JRLF	0.086±0.024a	0.583±0.229b	4.361±1.184a	8.961±2.533a	10.550±1.851a
0.10 g/ml JRLF	0.058±0.024ab	0.392±0.174b	5.022±2.004a	9.603±1.581a	10.561±1.296a
PSB	0.000±0.000b	0.022±0.022c	0.367±0.367d	1.544±0.953d	1.911±0.946d
0.01 g/ml JRP	0.333±0.187a	1.161±0.681bc	2.456±0.824d	3.219±1.004d	4.022±1.061d
0.03 g/ml JRP	0.075±0.024ab	5.203±0.763a	12.036±0.685c	11.697±0.715c	13.156±1.364c
0.06 g/ml JRP	0.275±0.155ab	2.767±0.789b	19.103±4.037b	18.719±2.413b	26.008±3.464b
0.10 g/ml JRP	0.081±0.047ab	2.983±0.621b	34.817±3.336a	49.564±3.602a	52.244±2.951a
PSB	0.000±0.000b	0.022±0.022b	0.367±0.367c	1.544±0.953c	1.911±0.946c
0.01 g/ml JRPF	0.025±0.012a	0.228±0.104b	0.531±0.109c	0.619±0.128c	0.864±0.180c
0.03 g/ml JRPF	0.014±0.009ab	0.236±0.073b	1.364±0.321bc	2.789±0.328c	4.308±0.384c
0.06 g/ml JRPF	0.008±0.008ab	0.358±0.077b	6.653±0.811b	11.139±1.868b	14.092±0.484b
0.10g/ml JRPF	0.022±0.006ab	1.686±0.407a	12.628±3.970a	27.444±4.205a	26.050±3.017a

^x^ Data shown are the means of two independent experiments, reported as the mean±standard error. Values followed by the same letters within the same column for the same media with different concentrations of rice tissue are not significantly different based on one-way ANOVA with least significant difference performed with SPSS at *P = 0*.*05*. Data were logarithm-transformed before analysis. The PSB was used as the control for the different rice tissue media.

DPI: days post incubation.

^y^ PSB: potato sucrose broth; JRL: Japonica rice leaf; JRLF: Japonica rice leaf filtrate; JRP: Japonica rice panicle; JRPF: Japonica rice panicle filtrate.

The conidia from 0.06 g/ml rice leaf media at 7 DPI were compared between rice cultivars Wanxian 98 and Huajing 952, and no significant difference was observed between IRL and JRL media (*P* = 0.677). To compare among different growth stage leaves, the first and second leaves from the top of rice cultivar Wanxian 98 at three or four days prior to the heading stage as well as leaves at the tillering stage were collected and used to make 0.06 g/ml media. No significant difference was observed among the different types of leaves (*P* = 0.795).

### Smaller conidia were produced in the rice tissue media

To investigate whether the conidia produced in the rice tissue media were similar to these produced in PSB, the conidial size (length and width) of strain G2 were investigated. Conidia produced in PSB and 0.06 g/ml rice tissue media (IRL, IRP, JRL and JRP) media were collected, and the length and width were measured. The results showed that the conidia produced in PSB were larger than these produced in IRL, IRP, JRL and JRP media (Table 3). These results indicated that even though the rice tissue media could stimulate the conidiation of *U*. *virens*, the conidia were smaller than these produced in PSB.

**Table 3:** Size of conidia of *U*. *virens* strain G2 produced in PSB and rice tissue media^x^.

	PSB^y^	IRL	IRP	JRL	JRP
Length (μm)	5.895±0.109a	5.017±0.087b	4.706±0.064c	5.166±0.078b	5.114±0.056b
Width (μm)	3.395±0.047a	3.262±0.032b	3.195±0.032b	3.090±0.028c	3.007±0.026c

^x^ Data shown are the means of the size of 100 conidia, reported as the mean±standard deviation. Values followed by the same letters within the same row are not significantly different based on one-way ANOVA with least significant difference tests performed with SPSS at *P = 0*.*05*.

^y^ PSB: potato sucrose broth; IRL: Indica rice leaf; IRP: Indica rice panicle; JRL: Japonica rice leaf; JRP: Japonica rice panicle.

### Germination was delayed for the conidia produced in rice tissue media

**Fig 1:**
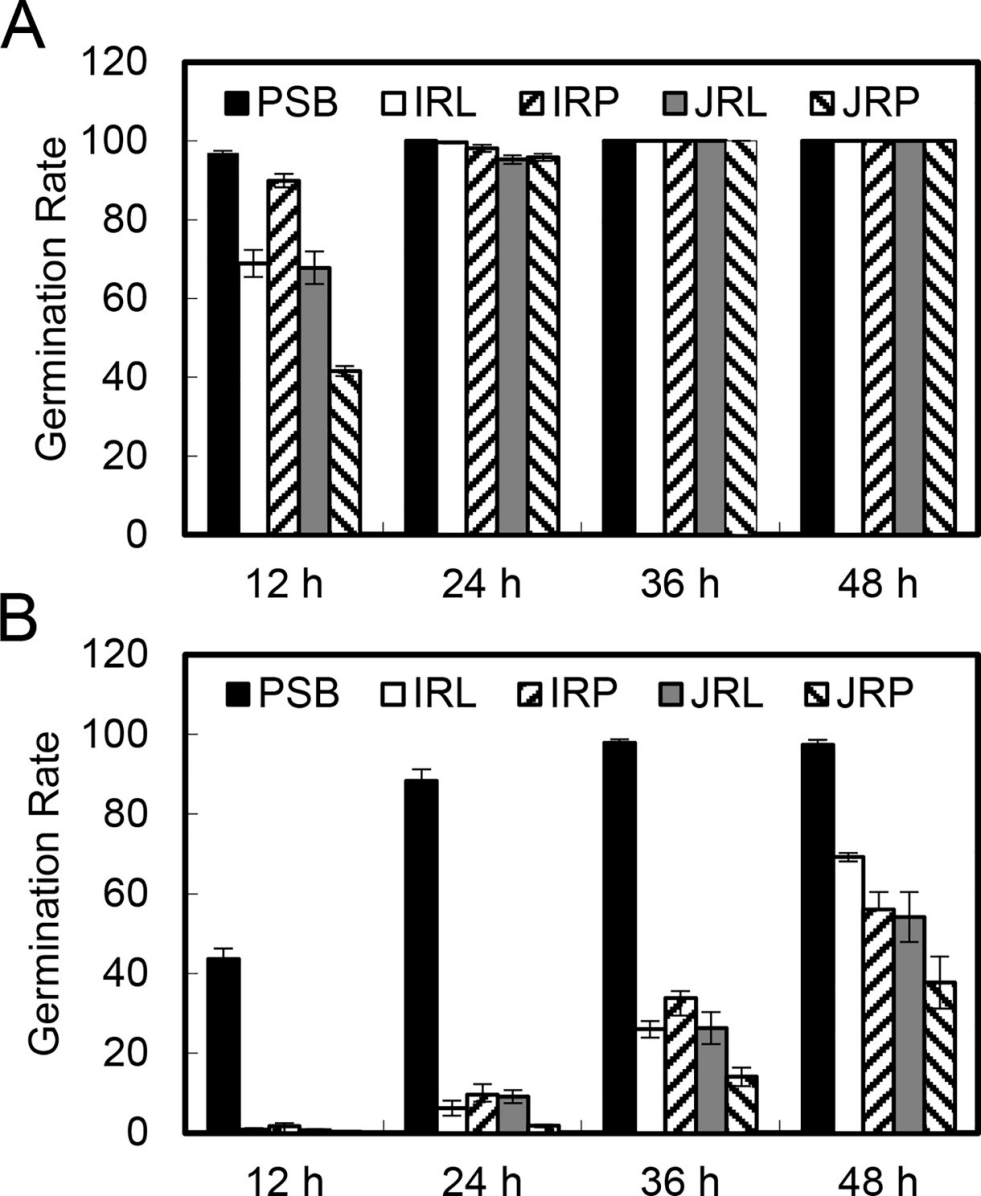
Conidia produced in rice tissue media have decreased germination rates. Conidia produced in 0.06 g/ml rice tissue media (IRL, IRP, JRL and JRP) and PSB were spread on PSA (**A**) or WA (**B**) plates and the germination rates were measured at 12 h, 24 h, 36 h and 48 h. Three independent experiments were performed, and similar results were obtained. Error bars represent the standard deviation of three replicates, and the different letters above each column indicate statistical significance (*P* < 0.05).

To investigate the germination of conidia produced in rice tissue media, conidial germination of strain G2 from 0.06 g/ml rice tissue media (IRL, IRP, JRL and JRP), as well as PSB were assessed on PSA and WA plates at 12, 24, 36 and 48 h. On PSA, the germination rate of conidia from PSB was 96.9% at 12 h. For conidia from IRL and JRL media, the germination rates were 68.9% and 67.8%, respectively. Conidia from IRP and JRP media showed the highest (89.9%) and lowest (41.6%) germination rates among these from the four different rice tissue media (Fig 1). After more than 24 h, almost all conidia had germinated. On WA, a germination rate of 43.6% was observed for conidia from PSB at 12 h, while lower germination rates were found for conidia from other media. Over time, the germination rate increased gradually and more than 90% of conidia from PSB germinated at 48 h. However, the germination rates were 69.2%, 56.1%, 54.2% and 37.7% for conidia from IRL, IRP, JRL and JRP media, respectively, which were significantly lower than that from PSB (Fig 1). These results showed that the germination of conidia from rice tissue media was slower than that from PSB, and generally the germination rate was higher on PSA than on WA.

### The mycelial mass was thin on rice tissue media

**Fig 2:**
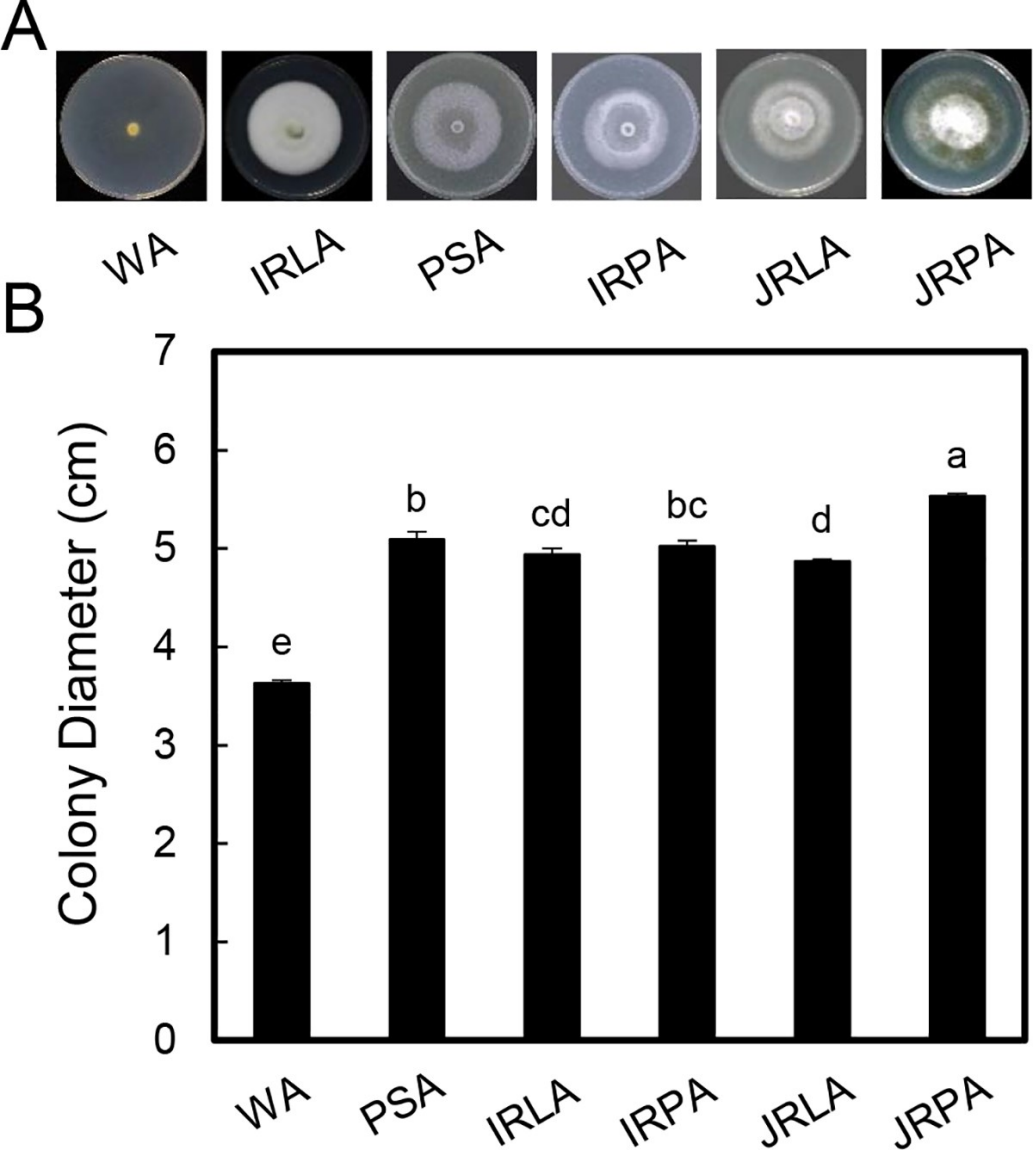
Rice tissue media do not affect hyphal growth. Plugs of strain G2 were inoculated on PSA, WA and 0.06 g/ml rice tissue media plates. The colonies were photographed (**A**), and their diameters (**B**) were measured for 3 weeks after inoculation. Three independent experiments were performed, and similar results were obtained. Error bars represent the standard deviation of three replicates, and the different letters above each column indicate statistical significance (*P* < 0.05).

Considering that rice tissue media promotes the production of conidia, we investigated whether the media provide enough nutrients for *U*. *virens* growth. Mycelial growth of strain G2 was assessed on PSA, WA and 0.06 g/ml rice tissue media solidified with 2% agar plates. The results showed that the colony diameter was largest on JRP media with agar (JRPA). On WA, though some hyphae were observed, they grew slowly and loosely. Despite the fact that mycelial growth was stimulated on some rice tissue media, the hyphae were loose and mycelia were thin (Fig 2). These results showed that *U*. *virens* can grow on both rice tissue media and PSA, but the overall biomass is decreased on rice tissue media, suggesting that rice tissue media could not provide enough nutrients for mycelial growth.

### Pathogenicity test for the induced conidia

To investigate the pathogenicity of conidia produced in rice tissue media, conidia of strain G2 were cultured in 0.06 g/ml IRL medium for 7 days. The 1.5 ml liquid supernatant were centrifuged at 7000 rpm for 1 min for conidia collection. Three treatments were performed: (I) the conidia were collected and diluted with water, (II) the conidia were collected and diluted with PSB, and (III) the conidia were collected and cultured in 50 ml PSB for 8–12 h at 160 rpm, then collected by centrifugation at 7000 rpm for 1 min and diluted with PSB. The concentration was adjusted to 1.0×10^6^ conidia/ml for inoculation. The results showed that more smut balls were observed for treatment III, whereas few or no smut balls were observed for treatments I and II (Table 4). These results showed that the conidia produced in rice tissue media can germinate and infect rice successfully, they should be incubated for 8–12 h in PSB before they are used to inoculation.

**Table 4:** Pathogenicity testing of the different conidia treatments.

Treatment^x^	NO. of false smuts^y^
I	0.5±0.07b
II	0.1±0.3b
III	20.9±10.5a

^x^ Conidia from *U*. *virens* strain G2 from 0.06 g/ml IRL were collected, and then treated as follow: I, the conidia were collected and diluted with water; II, the conidia were collected and diluted with PSB; III, the conidia were collected and cultured in PSB for 8–12 h and collected again and diluted with PSB.

^y^ Data shown are the means of three independent experiments, reported as the mean±standard error. Values not followed by the same letter within a column differ significantly based on one-way ANOVA with least significant difference tests performed with SPSS at *P = 0*.*05*.

### Conidia of conidiation-defective isolates were induced in rice leaves medium

The 0.06 g/ml IRL medium was used to investigate whether conidiation could be induced in conidiation-defective isolates. The isolates D32-1, HWD2-2, UV-8, 09-11-1-1 and 09-14-21 were selected because all of them are failed to produce conidia or only produce few conidia. For all of these isolates, conidia were produced at 3 or 4 DPI in 0.06 g/ml IRL medium, with substantial amounts of conidia produced at 6 or 7 DPI; conversely, little or no conidia were observed in PSB. The isolates HWD2-2 and UV-8 produced the most conidia (up to 2.3×10^7^ conidia/ml) in the IRL medium, an amount equal to that produced by non-defective isolates in PSB. The other isolates, D32-1, 09-11-1-1 and 09-14-21, produced fewer conidia, but conidia production were still within the same order of magnitude as that produced by HWD2-2 and UV-8. These results indicated that the IRL medium could be used to induce conidiation in defective isolates (Table 5).

**Table 5:** Conidiation of defective isolates in Wanxian 98 rice leaf medium.

Strain	Medium^y^	Conidiation (×10^6^ conidia/ml)^x^				
		3 DPI	4 DPI	5 DPI	6 DPI	7 DPI
D32-1	PSB	0.000±0.000a	0.000±0.000a	0.000±0.000b	0.000±0.000b	0.000±0.000b
	0.06 g/ml IRL	0.000±0.000a	0.133±0.088a	3.056±1.188a	3.033±0.290a	3.753±0.453a
HWD2-2	PSB	0.000±0.000a	0.000±0.000b	0.000±0.000b	0.000±0.000b	0.000±0.000b
	0.06 g/ml IRL	0.436±0.404a	10.606±2.408a	18.094±2.124a	25.600±1.785a	23.225±1.840a
UV-8	PSB	0.000±0.000a	0.000±0.000a	0.014±0.009b	0.028±0.018b	0.019±0.008b
	0.06 g/ml IRL	0.022±0.022a	0.661±0.394a	6.197±1.956a	16.972±1.084a	23.900±2.152a
09-11-1-1	PSB	0.000±0.000a	0.000±0.000b	0.006±0.004b	0.008±0.006b	0.003±0.003b
	0.06 g/ml IRL	0.597±0.236a	2.244±0.666a	3.583±0.427a	3.500±0.300a	3.300±0.435a
09-14-21	PSB	0.000±0.000a	0.000±0.000a	0.000±0.000b	0.000±0.000b	0.003±0.003b
	0.06 g/ml IRL	0.108±0.086a	0.819±0.339a	3.044±0.948a	3.639±1.045a	4.317±1.143a

^x^ Data shown are the means of two independent experiments, reported as the mean±standard error. Values followed by the same letter within a column for the same strain are not significantly different based on Student’s *t*-tests with *P = 0*.*05*.

DPI: days post incubation.

^y^ PSB: potato sucrose broth; IRL: Indica rice leaf.

## Discussion

In this study, rice leaves and panicles were crushed to make rice tissue media that could promote the conidiation of *U*. *virens* within a shorter timeframe than the PSB medium. Rice leaves medium was especially useful for the production of conidia in conidiation-defective strains. Our experiment showed that rice tissue media were effective in inducing conidia of *U*. *virens*.

Conidia play an important role in the process of rice smut infection and is essential for rice inoculation [7, 18, 25, 27]. For *U*. *virens*, temperature, medium and strain are important for conidia production. Currently, PSB medium were commonly used for conidia production [18, 33, 36, 37, 39]. However, conidiation varies among different isolates and conidia production can be unstable, eventually receding along with the increased numbers of transfer or long-term storage. Researches showed that pine needle and mulberry leaf media can induce sporulation in plant pathogenic fungi [40], and different kinds of plant leaves can induce sporulation in *Colletotrichum dematium* [41]. A carrot medium was considered as the best medium for obtaining conidia from *Venturia nashicola* [42]. In addition, dried barley seeds were added to 2% sucrose-amended PDB medium to improve conidia production in *U*. *virens* [34]. Starvation or nutritional depletion often stimulates sporulation [43], but no clear research has been performed for *U*. *virens*. This is consistent with our results, considering less nutrition in rice tissue media.

The conidiation capacity of *U*. *virens* in rice tissue media was positively related to tissue concentrations, except for 0.1 g/ml rice leaf media in which insufficient shaking occurred. Meanwhile, the media with rice leaf or panicle filtrates induced fewer conidia compared with unfiltered media, probably because the residues contain substances or provide certain physical benefits that affect conidiation. Less conidia were produced in 0.01 g/ml JRPF medium than in PSB medium, which might be caused by insufficient nutrients. There is no significant differences for conidia production between at 0.06 g/ml IRL and IRP medium. However, 0.06 g/ml JRL medium induced more conidia than JRP medium. No differences were observed for conidia production between different rice varieties leaf media or different growth stages of rice. However, for the filtered leaves, rice cultivar Wanxian 98 was more efficient to induce conidiation than Huajing 952. The possible reason could be some kinds of substance was filtered which was important for conidiation. For all the media, conidia production was stable in different batches and different tissue concentrations based on multiple independent experiments. Although 0.10 g/ml panicle was most efficient for conidiation, considering that the leaves are easily available and leaf medium also were able to induce sufficient conidia, therefore 0.06 g/ml leaf medium is recommend for pathogenicity test, inducing conidia and other experiments, such as protoplast preparation.

The conidia produced in rice tissue media were smaller than these produced in PSB medium, and the germination rates of the conidia from rice tissue media were lower than these produced in PSB on WA plates, indicating that sufficient nutrition is important for germination of conidia and the conidial growth, but it still has disease-causing potential. Conidia produced in rice leaf media and then cultured in PSB for 8–12 h with shaking were able to infect rice. However, conidia produced in rice leaf media without cultured in PSB lost the ability to infect rice, even they were diluted in PSB. For successful inoculation, some studies showed the conidia have to be diluted or resuspended in PSB [26, 27]. These results indicated that conidia produced in rice tissue media do not have enough nutrition and should be further cultured in PSB to achieve successful infection. For *U*. *virens* strains, the conidiation is easily to degeneration or loss after long-term storage or transfer many times. The rice tissue medium was able to induce conidia production for the conidiation-defective isolates. Although it need be cultured in PSB for several hours before inoculation, our researches provide an excellent approach to solve these kinds of problems.

In conclusion, we developed rice tissue media that were more effectively increase conidiation of *U*. *virens* within a shorter timeframe than the PSB medium. These media are valuable because many important isolates frequently lose their ability of conidiation after many transfers or long-term storage. Our media could stimulate and recover conidiation of such isolates, thus providing a solid foundation for continuously performing pathogenicity testing, which is necessary for genetic analyses of rice resistance and fungal virulence test.
