# Correction: Development of rice conidiation media for *Ustilaginoidea virens*

**DOI:** 10.1371/journal.pone.0227426

**Published:** 2019-12-30

**Authors:** Yufu Wang, Fei Wang, Songlin Xie, Yi Liu, Jinsong Qu, Junbin Huang, Weixiao Yin, Chaoxi Luo

In Fig 2A, the labels IRLA and PSA are swapped in the second and third image. The second image should be PSA and the third should be IRLA. Please see the correct Fig 2 here.

**Fig 2:**
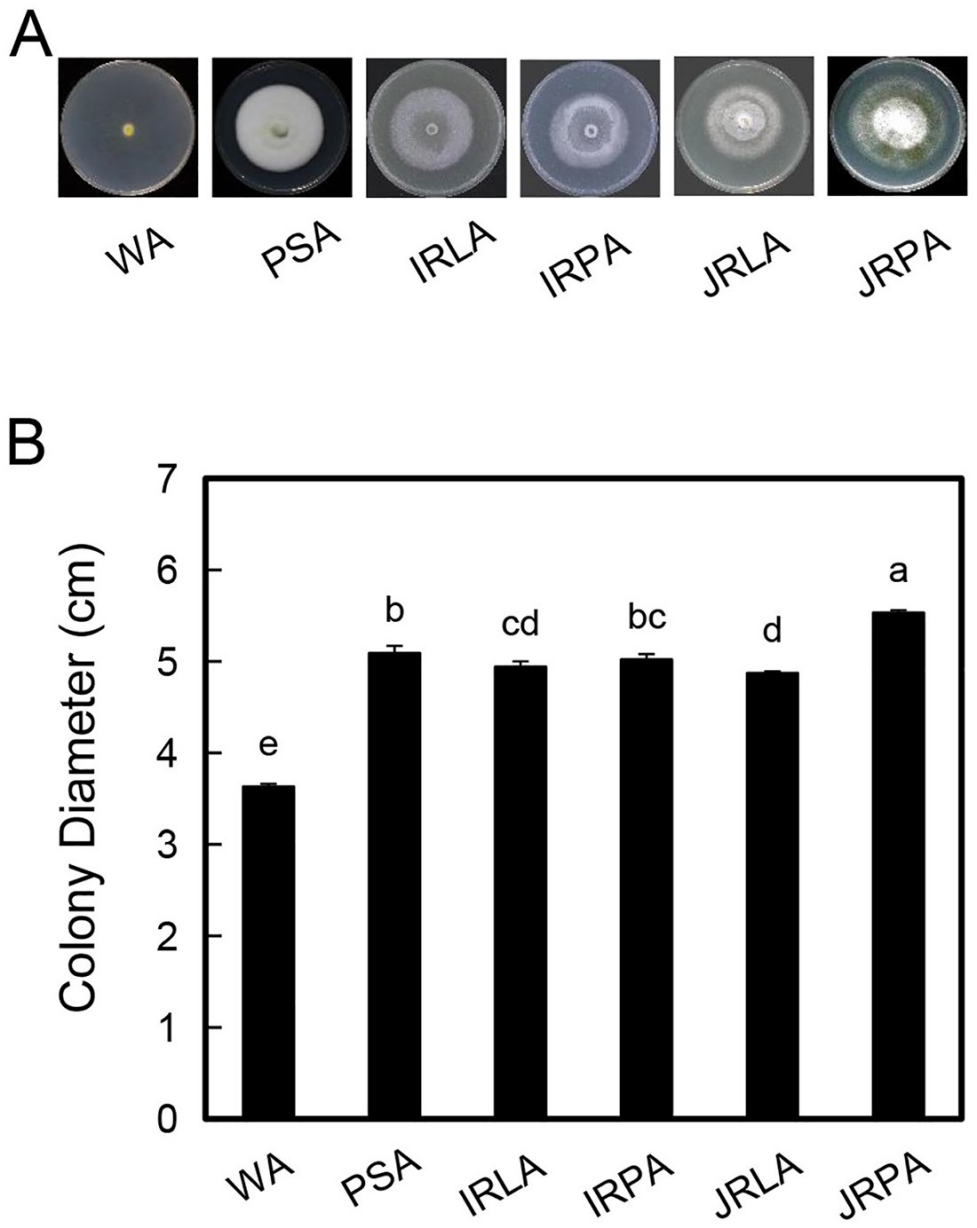
Rice tissue media do not affect hyphal growth. Plugs of strain G2 were inoculated on PSA, WA and 0.06 g/ml rice tissue media plates. The colonies were photographed (A), and their diameters (B) were measured for 3 weeks after inoculation. Three independent experiments were performed, and similar results were obtained. Error bars represent the standard deviation of three replicates, and the different letters above each column indicate statistical significance (P < 0.05).
